# Flavonoid Composition of *Salacia senegalensis* (Lam.) DC. Leaves, Evaluation of Antidermatophytic Effects, and Potential Amelioration of the Associated Inflammatory Response

**DOI:** 10.3390/molecules24142530

**Published:** 2019-07-10

**Authors:** Nelson G. M. Gomes, Andreia P. Oliveira, Diana Cunha, David M. Pereira, Patrícia Valentão, Eugénia Pinto, Luísa Araújo, Paula B. Andrade

**Affiliations:** 1 REQUIMTE/LAQV, Laboratório de Farmacognosia, Departamento de Química, Faculdade de Farmácia, Universidade do Porto, R. Jorge Viterbo Ferreira, nº 228, 4050-313 Porto, Portugal; 2 Laboratório de Microbiologia, Departamento de Ciências Biológicas, Faculdade de Farmácia, Universidade do Porto, R. Jorge Viterbo Ferreira, nº 228, 4050-313 Porto, Portugal; 3 Centro Interdisciplinar de Investigação Marinha e Ambiental (CIIMAR/CIMAR), Universidade do Porto, Edifício do Terminal de Cruzeiros do Porto de Leixões, Av. General Norton de Matos s/n, 4450-208 Matosinhos, Portugal; 4 MDS—Medicamentos e Diagnósticos em Saúde, Avenida dos Combatentes da Liberdade da Pátria, Bissau, Guiné-Bissau

**Keywords:** antifungal, flavonoids, inflammation, Isoquercitrin, Myricetin-3-*O*-rhamnoside, Quercetin-3-*O*-xyloside, Quercitrin, traditional medicine

## Abstract

Predominantly spread in West Tropical Africa, the shrub *Salacia senegalensis* (Lam.) DC. is known because of its medicinal properties, the leaves being used in the treatment of skin diseases. Prompted by the ethnomedicinal use, a hydroethanolic extract obtained from the leaves of the plant was screened against a panel of microbial strains, the majority of which involved in superficial infections. The extract was found to be active against the dermatophytes *Trichophyton rubrum* and *Epidermophyton floccosum*. Notable results were also recorded regarding the attenuation of the inflammatory response, namely the inhibitory effects observed against soybean 5-lipoxygenase (IC_50_ = 71.14 μg mL^−1^), no interference being recorded in the cellular viability of RAW 264.7 macrophages and NO levels. Relevantly, the extract did not lead to detrimental effects against the keratinocyte cell line HaCaT, at concentrations displaying antidermatophytic and anti-inflammatory effects. Flavonoid profiling of *S. senegalensis* leaves was achieved for the first time, allowing the identification and quantitation of myricitrin, three 3-*O*-substituted quercetin derivatives, and three other flavonoid derivatives, which may contribute, at least partially, to the observed antidermatophytic and anti-inflammatory effects. In the current study, the plant *S. senegalensis* is assessed concerning its antidermatophytic and anti-inflammatory properties.

## 1. Introduction

As part of our ongoing research program on plants from the Afrotropical realm [1,2,3], the antimicrobial properties of *Salacia senegalensis* (Lam.) DC. were investigated; this involved being subjected to a phytochemical study. Commonly known in Guinea-Bissau as “*mancuba*” or “*mancúbaru*”, *S. senegalensis* (Celastraceae) is a shrub which is widely spread in West Tropical Africa, particularly in Guinea-Bissau, Senegal, and Guinea [4]. Predominantly known for its antimalarial effects, extracts from the leaves are also reported to be used in the treatment of skin malignancies in Guinea and Nigeria [5,6,7].

Prior meta-analyses demonstrated a higher hit rate for antifungal activity with plants recorded for their ethnomedicinal use in the treatment of skin infections with an obvious pathological expression, in comparison with “random” collection [8]. Consequently, a hydroethanolic extract obtained from the leaves of *S. senegalesis* was selected and evaluated for its efficacy against a panel of fungi responsible for superficial infections. As a result of their high incidence, the panel also included fungal strains involved in systemic infections and the bacterial pathogens *Staphylococcus aureus* and *Escherichia coli*. This study was also motivated by the increasing incidence of skin microbial infections [9]. For example, dermatophytes affect 20–25% of the world population, with non-dermatophyte molds and yeasts further contributing to a public health problem related with skin pathogens, which translates into an economic burden [9,10]. While *Candida albicans* is the most common opportunistic human fungal pathogen [10], tinea pedis and onychomycosis are becoming an epidemiological problem, the anthropophilic species *Trichophyton rubrum* being the main etiological agent worldwide [9]. 

The assessment of the antimicrobial effects upon exposure to the extract obtained from the leaves of *S. senegalensis* was followed by assays geared towards the evaluation of its interference with RAW 264.7 macrophages and the enzymatic activity of the eicosanoid-metabolizing enzyme 5-lipoxygenase (5-LOX). Macrophages are one of the pivotal elements in the control of dermatomycotic infections, being involved in the microcidal effects, particularly through the production of reactive oxygen species (ROS) and reactive nitrogen species (RNS) [9,11]. While RNS appear not to be involved in the inflammatory response to specific fungal infections, such as dermatophytic ones, most skin pathogens are keratinophilic (i.e., produce keratinolytic enzymes) and are responsible for inducing an inflammatory reaction at the site of the infection [12]. Particularly concerning common dermatomycotic agents, the stepwise process of host infection is similar among most of the dermatophytes, as well as *Candida* spp. and *Malassezia* spp., including an inflammatory response mediated by eicosanoid-metabolizing enzymes [9,12].

As reviewed by Bagnazari et al. [13], several *Salacia* spp. have been reported to display relevant biological properties, namely antioxidant, anti-inflammatory, and antimicrobial activities; it is worth highlighting the antidiabetic properties of the Ayurvedic herbal medicine *Salacia reticulata* attributed to the highly potent α-glucosidase inhibitor salacinol and related sulfonium-ion inhibitors [14,15]. As there are only a few studies dealing with the biological properties of *S. senegalesis*, in the current study we mainly aimed to evaluate the potential of its leaves on the treatment of skin infections. As such, the hydroethanolic extract obtained from the plant was evaluated for its antimicrobial effects as well as its potential interference with pivotal targets that mediate the inflammatory response derived from cutaneous infection. Cytotoxicity against the skin cell model HaCaT cells was also investigated.

## 2. Results and Discussion

### 2.1. Characterization of the Flavonoid Profile of S. senegalensis Leaves

**Figure 1:**
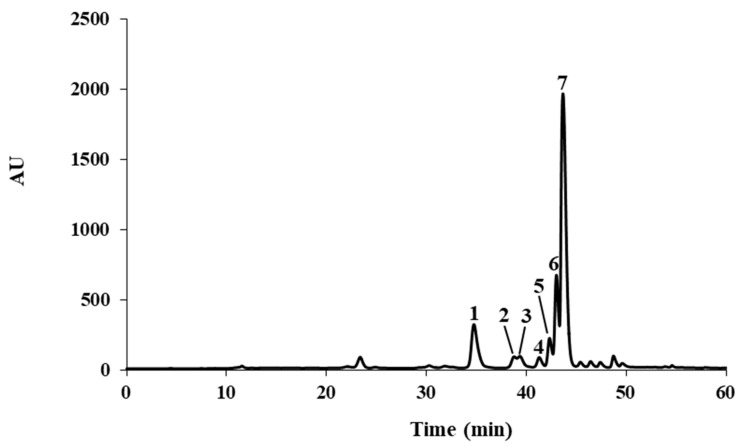
HPLC-UV (350 nm) profile of the hydroethanolic extract from *Salacia senegalensis* leaves. Identity of compounds **1**–**7** as in Table 1 and text.

**Figure 2:**
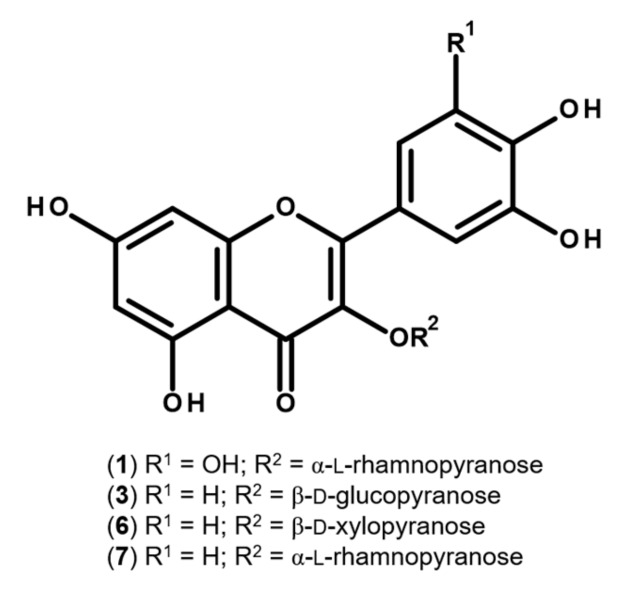
Structure of myricitrin (**1**), and quercetin 3-*O*-glycosides isoquercitrin (**3**), quercetin-3-*O*-xyloside (**6**) and quercitrin (**7**).

While being a species widely distributed in West Tropical Africa, reports on the chemical profile of *S. senegalensis* are scant. Previous studies on the leaves of the plant include the analysis of the proximate, vitamin and mineral composition [6], and the chemical profiling of its essential oil [5]. Adumanya [16] reported a series of phenolic acids in leaves from *S. senegalensis* obtained from an undefined location, details on the preparation of the extracts and chromatographic conditions also being absent. As such, because of the paucity of information on the metabolic versatility of the plant, the chemical profile of the leaves was investigated. Comparison with authentic substances did not allow us to identify any of the previously reported phenolic acids on the hydroethanolic extract obtained from the leaves of *S. senegalensis* under study. On the other hand, HPLC-DAD analysis allowed the identification and quantitation of several flavonoids: myricetin-3-*O*-rhamnoside (myricitrin; **1**), three quercetin derivatives (**3**, **6,** and **7**), and other flavonoids (**2**, **4,** and **5**) (Figure 1 and Table 1). Comparison with authentic standards allowed us to unequivocally ascribe **1** as myricitrin, as well as **3**, **6,** and **7** as the mono-*O*-glycosyl quercetin derivatives quercetin-3-*O*-glucoside (isoquercitrin), quercetin-3-*O*-xyloside, and quercetin-3-*O*-rhamnoside (quercitrin), respectively (Figure 2).

The UV spectra exhibited by compounds **2**, **4,** and **5** clearly indicated that they are flavonoid derivatives, namely 3-*O*-substituted quercetin derivatives (Table 1), characterized by a hypsochromic shift of *ca.* 20 nm (372 → 355) in band I and a reduction in intensity relative to band II, in comparison with the aglycone quercetin (*λ*max 255, 295sh and 372 nm) [17]. Therefore, considering the UV data and the presence of other quercetin derivatives, these compounds are also probably quercetin glycosides.

Quantitatively, the hydroethanolic extract obtained from *S. senegalensis* leaves was found to be rich in flavonoids, exhibiting a high content of quercetin derivatives (81.8%), quercitrin (**7**) being identified as the main constituent (68.6%), followed by myricitrin (**1**), constituting *ca.* 12.7% of the determined flavonoids (Table 1).

### 2.2. Antimicrobial Effects

While predominantly known for their antidiabetic properties, several species belonging to the genus *Salacia* have been reported to display antimicrobial properties, particularly against bacterial pathogens [18,19,20]. In contrast, reports on the antidermatophytic effects are restricted to a study with an ethanolic extract obtained from the bark of *Salacia crassifolia*, proving to be active against *T. rubrum* and *Trichophyton mentagrophytes* [21].

As in Svetaz et al. [8] and following a standardized definition of “*active*” extracts, only minimum inhibitory concentration (MIC) values ≤ 1 mg mL^−1^ were considered relevant. As seen in Table 2, the hydroethanolic extract obtained from the leaves of *S. senegalensis* was found to be active against the anthropophilic dermatophytes *T. rubrum* and *Epidermophyton floccosum* (MIC = 1 mg mL^−1^), proving it to be a fungicidal agent, exhibiting minimum lethal concentration (MLC) values of 2 and 4 mg mL^−1^, respectively. It is also worth noting the moderate fungistatic effects (MIC = 2 mg mL^−1^) against the zoophilic dermatophytes *Microsporum canis* and *T. mentagrophytes*, while it was less effective against the geophilic *Microsporum gypseum* (MIC = 4 mg mL^−1^) (Table 2). The extract, at 4 mg mL^−1^, was unable to exert fungistatic/fungicidal effects against the remaining human pathogenic fungi (Table 2). Although without relevant antibacterial effects (Table 2), a bacteriostatic effect was observed for *S. aureus* at 2 mg mL^−1^, a species frequently associated with dermatophytes in superficial infections [22].

While commonly mimicking other clinical conditions [12], the most common dermatophyte-derived lesions exhibit evident clinical signals, characterized as circular and erythematous, being easily detected and followed by traditional healers [8]. Curiously, the hydroethanolic extract obtained from the leaves of *S. senegalensis* was found to be solely active against causal agents related to skin or mucosal infections characterized by an evident and visible pathological expression, such as the dermatophytes *T. rubrum* and *E. floccosum*, while being devoid of notable fungicidal or fungistatic effects against yeasts or *Aspergillus fumigatus* (Table 2).

Flavonoids have been reported as a privileged class of antifungal agents due to their ability to interfere with several cellular targets [23]. Flavonols, in particular, have received the most attention due to the wide spectrum of associated antifungal effects, as well as their ability to suppress a number of fungal virulence factors [23,24]. Previous reports on the fungitoxic effects of isoquercitrin (**3**) evidenced a strong fungistatic effect against a strain of *T. rubrum* obtained from the Korean Collection for Type Cultures (KCTC), with a MIC value of 2.5 μg mL^−1^ [25]. Relevantly, structure–activity relationship (SAR) studies revealed that flavonoids with free C-5 and C-7 hydroxyl groups in ring A, such as those found in the extract under study (Table 1 and Figure 2), tend to be particularly active against *T. rubrum* [26]. Since there are no reports on the effect of myricitrin (**1**) and quercitrin (**7**) against *T. rubrum*, both flavonols were individually assessed at concentrations as high as 50 and 200 μg mL^−1^, respectively. No noticeable fungitoxicity was observed. However, exposure to a mixture of myricitrin (**1**) and quercitrin (**7**) at concentrations of 10 and 55 μg mL^−1^ (corresponding to the MIC value recorded upon treatment with *S. senegalensis* leaf extract) led to weak inhibitory effects (< 50%) against *T. rubrum*. As such, it is plausible to consider that the fungistatic effect observed against *T. rubrum* upon treatment with the extract under study (Table 2) may be partially related with a synergistic or additive effect between the identified flavonols.

Concerning the inhibitory effects against the dermatophyte *E. floccosum*, to the best of our knowledge, there are no reports on the fungitoxic effects of the flavonols identified in the *S. senegalensis* leaf extract. Similarly to what has been observed against *T. rubrum*, while none of the main components (**1** and **7**) identified in the extract were active against *E. floccosum*, a noticeable fungitoxic effect was observed upon exposure to a mixture of myricitrin (**1**) and quercitrin (**7**) at 10 and 55 μg mL^−1^, respectively.

### 2.3. Inhibitory Effects Against 5-Lipoxygenase Activity

The inflammatory response triggered by the presence of the fungus or the release of its metabolites and virulence factors play a relevant role in tissue damage during fungal infection [12,27]. In contrast to pathogenic yeasts, such as *C. albicans*, which generally leads to a low inflammatory response, dermatophytes frequently induce a significant level of inflammation and tissue damage [12]. The signs and symptoms elicited by inflammation, such as erythema, burning, or pruritus, are associated with most dermatomycotic infections [12]. While 5-LOX metabolism does not seem to play a key role in normal epithelial homeostasis, leukotrienes have long been associated with several inflammatory skin disorders [28,29]. 5-LOX from epidermal keratinocytes is involved in the production of the potent itch mediator leukotriene B_4_ (LTB_4_), being closely associated with dermatophyte-derived pruritus, the most common functional symptom of dermatophytosis [28]. In fact, the anti-inflammatory effects of conventional antifungal agents, such as several azoles, occur *via* the inhibition of 5-LOX, through the blockage of LTB_4_ synthesis in the skin [30].

**Figure 3:**
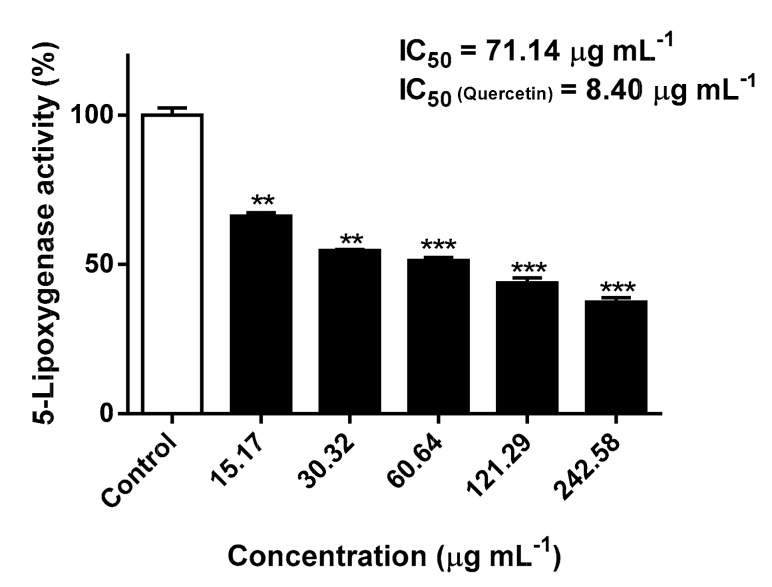
Inhibitory effects against 5-lipoxygenase (5-LOX) activity upon exposure to *S. senegalensis* leaf hydroethanolic extract. The results correspond to the mean ± SEM of three independent experiments performed in triplicate (statistical significance: ** *p* < 0.01 and *** *p* < 0.001).

Most structural research on the pharmacological interruption of the 5-LOX pathway has been done on soybean 5-LOX due to the lack of sufficiently purified human isoform (this is also due to the similarity between the active sites of human and soybean 5-LOX) [31]. In fact, the homology between human and soybean 5-LOX has long been known, as evidenced by cDNA sequencing [31,32]. Since the stereo- and regiospecific catalyzed incorporation of molecular oxygen into polyunsaturated fatty acids containing a (*cis*,*cis*)-1,4-pentadiene system is common between different isoforms of the enzyme [33], 5-LOX from *Glycine max* was used to evaluate the inhibitory effects of the *S. senegalensis* leaf extract. While displaying a lower inhibitory potency than the reference inhibitor quercetin (IC_50_ = 8.40 μg mL^−1^), a significant concentration-dependent inhibition of 5-LOX was observed at concentrations ranging from 15.17 to 242.58 μg mL^−1^ (*p* < 0.05): an IC_50_ value of 71.14 μg mL^−1^ was recorded (Figure 3).

In order to investigate if the observed inhibitory effects were related with the flavonols identified in the extract (Table 1 and Figure 2), the main components, myricitrin (**1**) and quercitrin (**7**), were evaluated for 5-LOX inhibition at concentrations corresponding to the IC_50_ value and the highest concentration of the extract. At the highest concentrations tested, both myricitrin (**1**) and quercitrin (**7**) were able to significantly inhibit the enzymatic activity of 5-LOX (*p* < 0.01) at 2.47 and 13.26 μg mL^−1^, down to 87.6 and 82.8%, respectively. However, at the concentrations corresponding to the IC_50_ value calculated after exposure to the extract obtained of *S. senegalensis* leaves, only **7** proved to be active, leading to a significant inhibitory effect (*p* < 0.01) at 3.89 μg mL^−1^ (8.68 μM), with 12.4% of inhibition. Our data corroborate previous findings on the inhibitory effects of both flavonols against 5-LOX [34,35]. In fact, not only the main components **1** and **7** have been previously reported as 5-LOX inhibitors, but also other flavonol derivatives identified on the hydroethanolic extract obtained from the leaves of *S. senegalensis* (Table 1 and Figure 2). Using the same *in vitro* model, Kim et al. [36] reported the inhibitory effects of isoquercitrin (**3**) and quercetin-3-*O*-xyloside (**6**) on 5-LOX, exhibiting IC_50_ values of 40.1 and 32.9 μM, respectively.

From these data, it is plausible to deduce a relationship between the flavonol content of the hydroethanolic extract from *S. senegalensis* leaves and the inhibitory effects on 5-LOX. In fact, SAR studies suggest that the flavonol series, particularly myricetin and quercetin derivatives, is a privileged group for the inhibition of the enzyme due to their chemical scaffolds [33,37]. Comparison between a series of structural analogues evidenced that pivotal features for the inhibition of 5-LOX include the presence of the 2,3-double bond with a 4-oxo functionality in the C-ring, since it completes a conjugated binding system through all the ring, thus stabilizing complexes or radical intermediates [33]. Furthermore, it confers planarity, allowing a more readily intercalation between aromatic or heteroaromatic amino acid residues at the active site of the enzyme [33,38]. Relevantly, it has been previously reported that the fully planar structure is best realized with quercetin and its derivatives [38]. SAR studies also denote the substantial importance of a catecholic arrangement in ring B, as the 3’4’-vicinal diol grouping confers a high stability to the radical form and participates in electron delocalization [33]. Finally, glycosylation at position C-3 greatly lessens the inhibitory effect, suggesting that the higher hydrophilicity restrains the intercalation in the lipophilic active site of LOXs [37,38].

The observed inhibitory effects are particularly relevant in dermatophytic infections, since both 5-LOX from the host epidermal keratinocytes and from the dermatophyte can be targeted. Dermatophytes display the ability for both *de novo* and “*trans-species*” production of leukotrienes *via* LOX during infection, enhancing the acute inflammatory response, a possible link between leukotriene production and fungal growth being also reported [39].

Relevantly, while the poor bioavailability and rapid metabolism of flavonoids has been associated with weak efficacy after oral administration, the facilitated skin penetration and high potency as inhibitors of 5-LOX suggests their potential use against superficial inflammatory conditions [40].

### 2.4. Effects Against RAW 264.7 Cells’ Viability and NO Levels

While the exact sequence of events involved in the immunologic defense against dermatophytes is not clearly defined yet, macrophages constitute a prominent element of antidermatophyte defenses, inducing the activation of an adaptative response to control the infection [9]. Upon infection by dermatophytes, the secretion of several cytokines by macrophages is increased, oxidative burst leading to an augmented secretion of RNS and ROS in activated macrophages [9]. Generally, the immune defense against mycotic agents partially depends upon the oxidative killing involving ROS and RNS [11]. In fact, nitrosative stress plays a preponderant and beneficial role in the antifungal effect against *T. rubrum*, an increased production of NO being related with the antidermatophytic effect [41].

**Figure 4:**
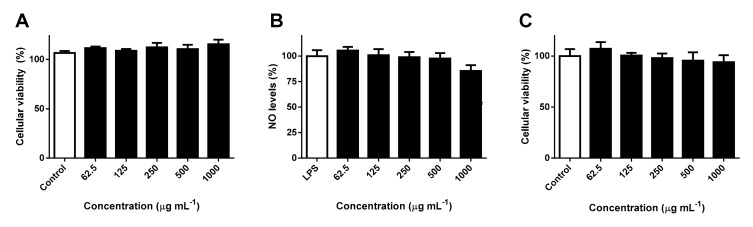
Effects of *S. senegalensis* leaf hydroethanolic extract on the cellular viability of (**A**) RAW 264.7 macrophages, (**B**) interference with NO levels in LPS-stimulated RAW 264.7 macrophages, and (**C**) on the cellular viability of HaCaT keratinocytes. The results correspond to the mean ± SEM of three independent experiments performed in triplicate.

Since the extract obtained from the leaves of *S. senegalensis* was found to be particularly active against dermatophytic strains, the potential hazardous interference with the mitochondrial viability of RAW 264.7 macrophages and NO levels was investigated. RAW 264.7 cells were incubated with increasing concentrations of *S. senegalensis* leaf extract, no significant effects being observed up to concentrations as high as the MIC value (1 mg mL^−1^) (Figure 4A). The potential interference of *S. senegalensis* leaf extract on lipopolyssacharide (LPS)-induced production of NO in RAW 264.7 cells was evaluated at concentrations ≤ 1 mg mL^−1^, through a mainstay cell-based *in vitro* assay based on the Griess method. As seen in Figure 4B, pre-treatment with the extract did not cause any notable effect on NO levels, being innocuous and conveniently not interfering in the production of the preponderant RNS mediator of the antidermatophytic response.

### 2.5. Effects Against HaCaT Cells’ Viability

Being the most common cell type of the human epidermis, keratinocytes are the first line of defense against microbial pathogens and other external agents, contributing to the regulation of a variety of physiological and pathological processes [42,43]. In fact, keratinocytes not only act as a building block of the skin but play also an active part in immune response, inflammatory processes, and wound healing, being involved in an interplay with various dermal immunocompetent cells [43].

Appropriate cell lines to assess skin biocompatibility and cytotoxicity include human epidermal keratinocytes, monolayer cultures of HaCaT cells being one of the most conventional *in vitro* models to address topical safety [44]. The cytotoxicity of the hydroethanolic extract obtained from the leaves of *S. senegalensis* was evaluated for 24 h, no significant interference on the cellular viability of HaCaT cells being noted at concentrations ranging from 62.5 to 1000 μg mL^−1^ (Figure 4C). Such results indicate that the extract has a safe toxicological profile against epidermal keratinocytes at concentrations displaying antidermatophytic and anti-inflammatory effects.

## 3. Materials and Methods

### 3.1. Plant Material and Extraction

Leaves from *S. senegalensis* (Lam.) DC., growing in Ilha Formosa, Guinea-Bissau, were collected in April 2017. The plant name was checked with The Plant List (http://www.theplantlist.org/tpl1.1/record/kew-2481214?ref=tpl1; accessed January 28, 2019). A voucher specimen of the leaves (GB72) was deposited at the Laboratory of Pharmacognosy, Faculty of Pharmacy, University of Porto. Samples were air-dried, ground to fine powder (mean particle size ≤ 910 μm), the hydroethanolic extract being obtained through maceration of *ca.* 6 g of the powdered material with 500 mL of a mixture of ethanol (Fischer Scientific, Loughborough, UK) and water (4:1), under magnetic stirring (450 rpm) for 30 min. The resulting extract was filtered through Whatman^®^ grade 1 filtration paper (Sigma-Aldrich, St. Louis, MO, USA), concentrated to dryness under reduced pressure in a Büchi Labortechnik AG Rotavapor^®^ R-215 (Flawil, Switzerland), and kept at −20 °C, in the dark, until analysis. The extraction yield was 21.12%.

### 3.2. HPLC-DAD Characterization of S. senegalensis Leaf Hydroethanolic Extract

The hydroethanolic extract was analyzed on an analytical HPLC unit (Gilson Medical Electronics, Villiers le Bel, France), using a 250 *×* 4.6 mm, 5 μm, Spherisorb ODS2 80 Å RP-C18 column (Waters, Dublin, Ireland). The dried residue (50 mg mL*^−^*^1^) was redissolved in methanol LiChrosolv^®^ (Merck KGaA, Darmstadt, Germany), filtered through a 0.45 μM pore size polytetrafluoroethylene (PTFE) membrane (Millipore, Bedford, MA, USA), and analyzed in triplicate. The mobile phase consisted of two solvents: HCOOH 5% (A) and methanol (B), starting with 5% B and using a gradient to obtain 15% B at 3 min, 25% B at 13 min, 30% B at 25 min, 35% B at 35 min, 45% B at 39 min, 45% B at 42 min, 55% B at 47 min, 75% B at 56 min, and 100% B at 60 min. The flow rate was 0.9 mL min*^−^*^1^. Detection was achieved with an Agilent 1100 series diode array detector (DAD) (Agilent Technologies, Waldbronn, Germany). Spectral data from all peaks were accumulated in the range 200–700 nm and chromatograms were recorded at 350 nm. Quantitative analysis was performed using external calibration curves (Table 3), using the equation of linear regression (concentration *vs.* optical absorbance at 350 nm) of each standard.

Calibration curves were built with five concentrations of each standard and analyzed in triplicate, according to the range of concentrations found in the hydroethanolic extract. Myricitrin (**1**), isoquercitrin (**3**), and quercitrin (**7**) were quantitated against their own standard curves, compounds **2**, **4**–**6** being also quantitated as isoquercitrin (**3**). The phytochemical standards isoquercitrin, myricitrin, quercitrin, and quercetin-3-*O*-xyloside were purchased from Extrasynthese (Genay, France). Linearity was evaluated from the coefficients of determination (*R*^2^) of the regression curves; limits of detection (LOD) and quantification (LOQ) were determined from the residual standard deviation (*σ*) of the curves and the slopes (*S*): LOD = 3.3 *σ*/*S* and LOQ = 10 *σ*/*S*. Data were processed on the Clarity software system, version 5.04.158 (DataApex Ltd., Prague, Czech Republic).

### 3.3. Antimicrobial Activity Evaluation

#### 3.3.1. Microbial Strains and Media

The antifungal activity was evaluated against ten fungal strains: three yeasts (*C. albicans* ATCC^®^ 10231, a fluconazole-resistant *C. albicans* clinical isolate D5, *Malassezia furfur* clinical isolate P26 and seven filamentous fungi (*A. fumigatus* ATCC^®^ 204305, *Scopulariopsis brevicaulis* clinical isolate FF and five clinical dermatophyte strains *T. rubrum* FF5, *T. mentagrophytes* FF7, *M. canis* FF1, *M. gypseum* FF3, and *E. floccosum* FF9). Clinical isolates were obtained from a recurrent case of oral candidosis (*C. albicans* D5), from nails and skin (dermatophytes, *S. brevicaulis* FF and *M. furfur* P26), identified by Prof. Dr. Eugénia Pinto (Laboratory of Microbiology, Faculty of Pharmacy, University of Porto). *Candida krusei* ATCC^®^ 6258 was used for quality control. The antibacterial potential was evaluated against Gram-negative bacteria (*E. coli* ATCC^®^ 25922) and Gram-positive bacteria (*S. aureus* ATCC^®^ 25923).

To guarantee the purity, viability, and optimal growth, all strains were sub-cultured before each assay: *C. albicans*, *A. fumigatus,* and *S. brevicaulis* on Sabouraud dextrose agar (SDA, bioMérieux, Marcy l’Etoile, France) and incubated at 35 °C for 24 h, 48 h, and 4 days, respectively; *M. furfur* on SDA with 2% of tween (TW) 80 was incubated for 3 days at 35 °C; dermatophytes on Mycosel agar (MYC, Becton Dickinson, Sparks, MD, USA) were incubated for 5–7 days at 25 °C; *S. aureus* and *E. coli* on Mueller–Hinton agar (MHA, Liofilchem, Roseto degli Abruzzi, Italy) were incubated for 24 h, at 35 °C. RPMI-1640 broth medium, with L-glutamine and without NaHCO_3_ (Biochrom GmbH, Berlin, Germany), used on the evaluation of antifungal activity, was buffered with 0.165 mol L^−1^ of 3-(*N*-morpholino)-propanesulfonic acid (MOPS, Sigma-Aldrich, St. Louis, MO, USA), pH being adjusted to 7.0 ± 0.2 with 1 mol L^−1^ NaOH. For *M. furfur*, 2% of TW80 was added to RPMI. Mueller–Hinton broth 2 (MHB2, Becton Dickinson, Sparks, MD, USA) was used for the evaluation of antibacterial activity.

#### 3.3.2. Antimicrobial Susceptibility Testing by Broth Microdilution

The minimum inhibitory concentrations (MICs) and minimal lethal concentrations (MLCs) were used for determining the antimicrobial activity in agreement with the recommendations of the Clinical and Laboratory Standards Institute (CLSI) reference documents, with minor modifications: M27-A3 [45] and M38-A2 [46] for yeasts and filamentous fungi, respectively; M100-A25 [47] for bacteria.

A 100 mg mL^−1^ solution of the extract was prepared in dimethylsulfoxide (DMSO, Sigma-Aldrich, St. Louis, MO, USA). Two-fold serial dilutions were prepared within the concentration range 0.25–4 mg mL^−1^, in MHB2 for bacteria and RPMI for fungi, no interference of DMSO on the bacterial/fungal growth being noted. Sterile and disposable microtiter plates, with 96 flat bottom wells, were used to evaluate the susceptibility of the microorganisms to the sample. Equal volumes of cell suspension and sample dilutions were added in the wells.

Three controls were performed: a sterility control, a growth control, and a quality control, performed with an ATCC^®^ reference strain, gentamicin (Sigma-Aldrich, Seelze, Germany) and voriconazole (kindly provided by Pfizer, Main St., Cambridge, MA, USA) being used as reference antibacterial and antifungal agents, respectively.

The hydroethanolic extract and compounds were tested in duplicate and independently at least three times.

##### Antibacterial Susceptibility Testing

Cell suspensions were prepared from pure cultures on MHA/24 h in a sterile saline solution, adjusted to obtain a MacFarland standard of 0.5 at 530 nm, corresponding to 1–5 × 10^6^ cells mL^−1^. This initial suspension was diluted in MHB2 to obtain an inoculum suspension of 1–5 × 10^4^ CFU mL^−1^. Gentamicine (0.06–4 µg mL^−1^) was used as a quality control, with *E. coli* ATCC^®^ 25922, the obtained results being within the recommended limits defined by CLSI protocols; a MIC value of 0.5 mg L^−1^ was determined. The plates were incubated aerobically at 35 °C for 24 h. The MIC was determined as the lowest concentration at which no visible growth was observed. The MLC was assessed by spreading 10 µL of culture collected from wells showing no visible growth on MHA plates. The MLC was determined as the lowest concentration at which no colonies grew after 16–18 h incubation at 35 °C.

##### Antifungal Susceptibility Testing

Yeast cells suspensions were prepared from pure cultures on SDA/24 h, or SAB + TW80/3 days, in sterile saline solution and adjusted to MacFarland standard of 0.5 at 530 nm, corresponding to an initial suspension of 1–5 × 10^6^ cells mL^−1^. This initial suspension was diluted in RPMI, or RPMI + TW80, to obtain an inoculum of 1–5 × 10^3^ CFU mL^−1^. For filamentous fungi, a spore suspension was prepared from pure culture with spores in SDA (*A. fumigatus* and *S. brevicaulis*) or MYC (dermatophytes) in sterile saline with one drop of TW20 added. The cell density was adjusted by the spore count and diluted in RPMI to obtain the adequate inoculum (1–3 × 10^3^ CFU mL^−1^ for dermatophytes and 0.4–5 × 10^4^ CFU mL^−1^ for *A. fumigatus* and *S. brevicaulis*). Voriconazole (0.06–2 µg mL^−1^) was used as a quality control with *C. krusei* ATCC^®^ 6258; a MIC value of 0.5 mg L^−1^ was obtained. The results obtained were within the recommended limits defined by CLSI protocols. The plates were incubated aerobically at 35 °C during 48 h for *C. albicans* and *A. fumigatus*, 3 days for *M. furfur* and at 25 °C for 5–7 days for the dermatophytes.

MICs were determined as the lowest concentrations resulting in 100% growth inhibition, in comparison to the sample-free controls. The MLC was assessed by spreading 10 µL of culture collected from wells showing no visible growth on SDA, or SAB+TW80, plates. The MLC was determined as the lowest concentration at which no colonies grew after 48 h incubation at 35 °C for *C. albicans* and *A. fumigatus*, 3 days for *M. furfur,* and 7 days at 25 °C for dermatophytes.

### 3.4. Inhibition of 5-Lipoxygenase

The evaluation of the 5-LOX inhibitory activity was based on the oxidation of linoleic acid to 13-hydroperoxylinoleic acid, as described in Kachmar et al. [48]. The absorbance of the reaction mixture containing 20 μL of extract/compound, 200 μL of phosphate buffer (pH = 9), 20 μL of soybean 5-LOX (100 U), and 20 μL of linoleic acid (in ethanol) was determined at 234 nm, at 37 °C, for 3 min, in a Multiskan^TM^ GO microplate reader (Thermo Fisher Scientific Oy, Vantaa, Finland). LOX (Type V; EC 1.13.11.12) and linoleic acid were purchased from Sigma-Aldrich (St. Louis, MO, USA). Three independent assays were performed in triplicate and results were compared with those obtained upon treatment with quercetin (positive control), tested under the same conditions.

### 3.5. Interference with RAW 264.7 Macrophages

#### 3.5.1. RAW 264.7 Cells Culture and Viability

Interference with the mitochondrial activity of RAW 264.7 macrophages was evaluated as in Ferreres et al. [49]. Murine macrophage ATCC^®^ RAW 264.7 cells (LGC Standards S.L.U., Barcelona, Spain) were maintained in Dulbecco’s Modified Eagle Medium (DMEM) supplemented with 10% heat-inactivated foetal bovine serum (FBS) and 1% penicillin/streptomycin (10000 U mL^−1^), grown in a Toreuse model 2428 incubator (Saint Louis, MO, USA) at 37 °C, in a humidified atmosphere of 5% CO_2_. DMEM, FBS, and penicillin-streptomycin solution (Pen Strep) were from GIBCO, Invitrogen (Grand Island, NY, USA). RAW 264.7 cells were harvested by scraping and then suspended in flasks for growth.

Cellular mitochondrial activity was evaluated by the thiazolyl blue tetrazolium bromide (MTT; Sigma-Aldrich, St. Louis, MO, USA) reduction assay, using increasing concentrations of extract in DMEM. Cells were cultured in 96-well plates (2.5 × 10^4^ cells well^−1^) and allowed to attach for 24 h. After incubation with the hydroethanolic extract, MTT was added to each well, the plate being incubated for 90 min at 37 °C. Formazan crystals were then solubilized by the addition of a DMSO: isopropanol (3:1) mixture and quantified spectrophotometrically at 560 nm in a microplate reader. The results of cellular viability correspond to the mean ± SEM of three independent experiments performed in triplicate, being expressed as a percentage of the untreated control cells.

#### 3.5.2. Determination of NO Levels

Interference with LPS-activated NO levels was determined by measuring the nitrite accumulated in cell-free supernatants, *via* the Griess reaction. RAW 264.7 cells were cultured in 96-well plates (3.5 × 10^4^ cells well^−1^) for 24 h and then pre-treated with different concentrations of extract for 2 h. LPS (1 μg mL^−1^) from *E. coli* (Sigma-Aldrich, St. Louis, MO, USA) was then added and the plates were further incubated for 22 h. Absorbance was read at 540 nm. The results correspond to the mean ± SEM of three independent experiments performed in triplicate, being expressed as percentage of NO in cells exposed to LPS (positive control for NO production). Further details are as described in Ferreres et al. [49].

### 3.6. HaCaT Cells Culture and Viability

Human epidermal HaCaT keratinocytes were obtained from ATCC^®^ and maintained in DMEM medium supplemented with 10% FBS and 1% penicillin/streptomycin (10,000 U mL^−1^), and grown as monolayer at 37 °C in a 5% CO_2_ atmosphere. After three days the culture was approximately 80% confluent, cells being harvested with trypsin-EDTA 0.25% solution (GIBCO, Invitrogen, Grand Island, NY, USA). Then the cells were subcultured in 96-well plates (1.5 × 10^4^ cells well^−1^), grown until 80% confluence, and subsequently exposed to the extract for 24 h at 37 °C. Interference with the mitochondrial activity was studied 24 h later by the MTT reduction assay as described in Gomes et al. [50]. Viable cells were calculated as a percentage of the negative control cells set at 100%, results corresponding to the mean ± SEM of three independent experiments performed in triplicate.

### 3.7. Statistical Analysis

Statistical analysis was performed using GraphPad Prism 6.01 Software (San Diego, CA, USA). Grubbs’ test was used to detect and exclude outliers. Prior to the analysis, the data set was checked for normality of distribution using Shapiro–Wilk test, ensuring that all data follow a normal distribution. Unpaired student’s *t*-test with Welch’s correction was used to compare the existence of significant differences between treatment and control groups. Values of *p* < 0.05 were considered statistically significant.

## 4. Conclusions

This paper describes for the first time the flavonoid profile of *S. senegalensis*, demonstrating the occurrence of myricitrin and quercetin derivatives, known for their antifungal and anti-inflammatory properties.

Furthermore, the antidermatophytic effects of a hydroethanolic extract obtained from the leaves of the plant, as well as the potential attenuating effects on the inflammatory response associated with dermatophytic infections, are also demonstrated. The extract was more active against the dermatophytes *T. rubrum* and *E. floccosum*, common causal agents of persisting dermatophytosis. As a result of the significant inhibitory effects against soybean 5-LOX, it seems evident that the hydroethanolic extract obtained from the leaves of *S. senegalensis* exerts an anti-inflammatory effect, at least in part, *via* the inhibition of the enzyme. It was also found that it did not interfere with the cellular viability of RAW 264.7 macrophages and NO levels, thus not hampering the potential fungicidal effects associated with the production of RNS. Relevantly, the current work indicates that the antidermatophytic and anti-inflammatory effects are achieved without interference with the cellular viability of the skin representative HaCaT cells.

We demonstrated that the leaf extract, obtained using cheap and non-hazard solvents, constitutes a source of potential crude drugs or bioactives, thus valorizing *S. senegalensis*.

## Figures and Tables

**Table 1:** Content of flavonoids in the hydroethanolic extract of *S. senegalensis* leaves.

Peak	Compounds	UV (nm)	mg Kg^−1^ Dry Extract
**1**	Myricitrin	259, 298sh, 351	10165.70 ± 119.98
**2**	Flavonoid derivative	257, 266sh, 355	916.35 ± 85.12
**3**	Isoquercitrin	257, 266sh, 353	1096.10 ± 48.29
**4**	Flavonoid derivative	257, 267sh, 355	731.05 ± 38.75
**5**	Flavonoid derivative	256, 266sh, 294sh, 354	2741.27 ± 113.18
**6**	Quercetin-3-*O*-xyloside	256, 265sh, 294sh, 354	9424.36 ± 213.32
**7**	Quercitrin	260, 306sh, 352	54675.24 ± 1352.64
Total			79750.07 ± 1971.28

**Table 2:** Antimicrobial effects upon exposure to the hydroethanolic extract obtained from the leaves of *S. senegalensis* against human pathogenic fungi and bacteria.

Microorganism	MIC ^a^	MLC ^b^
Fungi		
*Candida albicans* ATCC^®^ 10231	>4	>4
*Candida albicans* D5	>4	>4
*Malassezia furfur* P26	4	>4
*Aspergillus fumigatus* ATCC^®^ 204305	>4	>4
*Scopulariopsis brevicaulis* FF	>4	>4
*Epidermophyton floccosum* FF9	1	4
*Microsporum canis* FF1	2	4
*Microsporum gypseum* FF3	4	>4
*Trichophyton mentagrophytes* FF7	2	4
*Trichophyton rubrum* FF5	1	2
Bacteria		
*Escherichia coli* ATCC^®^ 25922	>4	>4
*Staphylococcus aureus* ATCC^®^ 25923	2	>4

^a^ MIC—minimum inhibitory concentration; ^b^ MLC—minimum lethal concentration. MIC and MLC values expressed in mg mL^−1^.

**Table 3:** Linear regression equation analysis, LOD^a^ and LOQ^b^, for external standards.

Standard	Regression Equation			Linearity Range (µg mL^−1^)	LOD (µg mL^−1^)	LOQ (µg mL^−1^)
	Slope (*S*)	Intercept (*b*)	*R*^2^ (n=3)			
Myricitrin	44.893	831.50	0.998	25–400	4.78	14.49
Isoquercitrin	59.725	694.77	0.996	12.5–200	0.507	1.537
Quercitrin	48.9	1085.9	0.997	25–400	0.591	1.792

^a^ LOD = Limit of detection; ^b^ LOQ = limit of quantification.
